# “We Never Stop Singing”: The Dynamics of the Mental and Physical Health of Czech Religious Pastors during the COVID-19 Pandemic

**DOI:** 10.1007/s10943-023-01977-3

**Published:** 2024-01-16

**Authors:** Silvie Kotherová, Michal Müller, Lenka Jedličková, Jakub Havlíček, Tomáš Bubík

**Affiliations:** 1 Department of Sociology, Andragogy and Cultural Anthropology, Faculty of Arts, Palacky University Olomouc, CO-LAB Palacky University Olomouc, Křížkovského 511/10, 779 00 Olomouc, Czech Republic; 2 Department of Economic and Managerial Studies, Palacky University Olomouc, Olomouc, Czech Republic; 3 Department of Sociology, Andragogy and Cultural Anthropology, Palacky University Olomouc, Olomouc, Czech Republic

**Keywords:** COVID-19 pandemic in Czech Republic, Religious coping, Mental and physical health, Credibility enhancing displays, Ritual behavior

## Abstract

This study explores the dynamics of coping strategies of Czech religious leaders during a peak of the COVID-19 pandemic. An interpretative phenomenological analysis reveals that mental health among pastors is closely linked to a need to maintain community and social contact, while physical health is related to limitations upon ritual elements. In all narratives, the lived experience of mental health in the form of prosocial behavior is significantly prioritized despite the possibility of spreading infection. The analysis also shows that maintaining the community is closely linked to risky behaviors, which positively affected group and individual well-being.

## Introduction

Over the years of 2020 and 2021, as the COVID-19 pandemic spread around the world, religious groups underwent many changes and faced various challenges, such as fear of illness, the consequences of illness, a reduction in the number meetings, or even the complete closure of churches for an extended period. In the first week of September 2020, the Czech Republic ranked among the worst affected countries in Europe in terms of the number of new infections per million inhabitants. By the beginning of January 2021, the country had the highest new infections rate in the world (ECHO24, 2021). During this wave, 30,000 people died. The entirety of Czech society, including religious activities, was locked down for almost four months throughout this period of January through April, 2021. Although these measures help to mitigate spread of the virus, they can also harm individuals at a psychological level. Prolonged quarantine can cause stress, anxiety (Huremović, 2019), insomnia, frustration, and many other pitfalls affecting general health (Brooks et al., 2020). In religious groups, this stress could also be exacerbated by fears regarding the religious groups’ survival.

Thanks to long-term field work, we learned about an event that happened in September 2020. Despite the high prevalence of COVID-19 infection in the population, a group of senior evangelical pastors met and performed a communion ritual under both kinds. They drank communion wine from a chalice or small individual glasses. Half of them drank from the same chalice. Nine of the fifteen pastors who attended the ritual became infected with the disease of COVID-19 and a majority of those experienced a very severe form of the disease necessitating hospitalization. This case was the inspiration for our study, which aims to map the motivations and coping strategies of this religious group during the COVID-19 pandemic. This aim led us to formulate the following research questions:

### RQ1:

What are the coping strategies of a religious group of evangelical pastors during the COVID-19 pandemic?

### RQ2:

 How do pastors make sense of the tension between the levels of mental and physical health that shapes their coping context?

Meeting with the spiritual community is important for religious life. Community interaction provides church members with appropriate social support and functions as a mitigation against negative psychological states. Nonetheless, any social interaction during a pandemic can be a risk to physical health. For believers to navigate the dynamic of governmental measures, doubts about their faith amid ubiquitous death are not easy. It is the uniqueness of this situation, distinctly existential in nature and touching the very essence of being and belief in general, that led us to use the qualitative approach of interpretive phenomenological analysis (IPA) (Smith et al., 2009).

## Theoretical Background

### Religion Community as a Social Determinant of Health

Even though the COVID-19 pandemic substantially limited religious community activities, religious life did not stop completely. The prominent sociologist of religion, Émile Durkheim, writes, “A religion is a unified system of beliefs and practices relative to sacred things, that is to say, things set apart and forbidden—beliefs and practices which unite in one single moral community called a Church, all those who adhere to them” (Durkheim, 1995, p. 44). Durkheim’s assertion is that the role of the community in religion is as important as religious faith and ritual. Also, religious ideas reach their maximum intensity when all community members are together and in a direct relationship when the same thought or feeling unites them (Durkheim, 1995, p. 386–387). As such religion presupposes the meeting of individuals and their groups. Community meetings reduce the likelihood that the bonds between believers, which are an essential part of religion, will weaken (Greenfield & Marks, 2007). Given the nature of the religious group we study and the fact that we are primarily interested in social activities, Durkheim provides a theoretical framework as a possible main predictor of the functioning of a religious group and with it the individual well-being of its members.

However, community does not only have a role for religion, it can also play an important role in the well-being of the individual community members. Religion as a social determinant of health has been largely overlooked and understudied (Kawachi, 2020; Ransome, 2020). However, several studies point to a close relationship between religion and well-being (Ellison, 1991; Koenig, 2012). As Greenfield and Marks (2007) show, religious social identity mediates the associations between frequent religious service attendance and all three dimensions of subjective psychological well-being examined. This relationship was also confirmed by Chen and VanderWeele (2018) in a longitudinal study that examines a large group of adolescents. Compared with non-attendance, attending religious services at least weekly is associated with greater life satisfaction and a positive effect on various character levels.

Frequency of ritual participation is also positively related with social well-being and sense of community (Sohi et al., 2018). The positive prosocial effects on the self-reported health of pilgrims participating in one of the world’s largest rituals, the Prayag Magh Mela, are confirmed by the longitudinal research of Khan et al. (2014). Recent research also shows that religious factors are related to moderation of stress hormones (Ai et al., 2010), to long-term changes in brain structures implicated in depression (Hayward et al., 2012), and the activation of control and reward centers in the brain (Inzlicht & Tullett, 2010; Schjødt et al., 2008).

While those studies point to a beneficial relationship between religious participation and well-being, they do not address how the dynamics of religious groups might change when religious contact is kept to a minimum, as during a pandemic. A pandemic itself puts people under stress from a potential illness (Tybur & Lieberman, 2016), which increases the need for religious reassurance (Meza, 2020; Minton & Cabano, 2020). These limitations lead to various psychological problems (Huremović, 2019), but little is known about how believers cope with this problem of limited religious contact. As such, we can also monitor whether it is sufficient for believers to move to an online form of meeting (Sulkowski & Ignatowski, 2020), or if they are looking for additional ways to avoid individual and community crises caused by limited social contact.

The pastor plays a specific role in the community and also experiences these limitations differently than lay believers. The pastor’s role is primarily to keep the community vibrant and to consider interventions which enable the community to suffer as little as possible when the ability to congregate is limited. Nevertheless, we also need to see how these interventions affect the function, behavior, and individual well-being of both the pastor and the community. What crises might the leader of the religious group face? These are necessary factors to observe to gain a comprehensive understanding of the phenomenon of religion and its impact on individual well-being.

As Durkheim postulated, religion consists of several factors: belief, ritual, and community, which are the three main topics mentioned by respondents of this study. Therefore, it is essential to see how the interrelation of these factors has changed during the COVID-19 pandemic crisis and whether any of the elements are more inhibited than others during periods of lockdown restrictions.

### How big can the Change of Ritual be?

Sociologists and anthropologists posit that one of ritual’s primary functions is to support group solidarity (e.g., Durkheim, 1995; Rappaport, 1999). Some scholars maintain that social bonding is not an end in itself but a means to facilitate intragroup cooperation. Irons (2001, 2004), for example, argues that the primary advantage of religious behavior is that it promotes cooperation and solutions to collective action problems that humans have faced throughout their evolutionary history. Rituals and the gatherings around them can play a significant role not only for individuals’ well-being but also for the survival of the group itself (Sosis, 2000; Sosis & Bressler, 2003). Ritual thus acts as a mutual symbiosis between the individual and the group that is beneficial to both parties.

Anthropologist Roy Rappaport (1979) delineated the essential elements of the ritual: no obvious empirical goals; compulsion; literalism and rigidity; repetition, reiteration, redundancy; order and boundaries. According to some theories (Boyer & Liénard, 2006; Liénard & Boyer, 2006) and research (Lang et al., 2015), such ritualized behavior helps manage disturbing thoughts, thereby reducing the potential anxiety related to them. Any changes to these elements, under the pressure of a pandemic or otherwise, could provoke an individual well-being crisis and potential crisis in the religious group. The COVID-19 pandemic disrupted several domains within the complex of Rappaport’s ritual elements (Rappaport, 1979). The most disruptive factors are the impossibility of repeating the religious ritual in closed churches, the rigidity of standard ritual arrangements, and that sacred ritual boundaries were shifted into profane homes or virtual environments. The topics we follow in connection with these theories focus on how ritual has changed during the COVID-19 pandemic and which changes are most problematic for individual and group mental health.

### Costly Signaling and Credibility Enhancing Displays

It is important to realize that a group of pastors facing church closures and other restrictions might feel an overall threat to the functioning of their religious society. Moreover, they may feel the need to signal the importance of religious action and faith accessed through ritual activity. As some research shows, men in religious communities who felt threatened conducted costly painful rituals to promote group solidarity (Sosis et al., 2007). By drinking from the same goblet, the pastors within the community of senior pastors could signal to one another their cohesion at a time of great crisis in the church. Costly rituals do not necessarily involve pain but generally see practitioners’ making a significant investment, e.g., of time or money, to signal their commitment to cooperating with members of a particular group (Sosis, 2006) and a particular ideology or religious belief (Henrich, 2009). Thus, pastors can send signals within the pastor community but can also send signals to their community of believers by performing credibility enhancing displays. As the study by Singh and Henrich (2020) showed, religious believers are more likely to regard the self-denying religious representative (shaman) as a stronger believer. Practitioners’ making such investments in costly practices interlocked with beliefs becomes a self-stabilizing mechanism that helps sustain the group (Henrich, 2009). As some research has shown, participating in rituals promotes intragroup cooperation, allowing religious communal societies to survive longer than their secular equivalent (Sosis, 2000; Sosis & Bressler, 2003). Richard Sosis (2000) showed on dataset of two hundred nineteenth-century US communal societies that religious communes are between two and four times more likely to survive in every year of their life course than their secular counterparts.

These results suggested a strong relationship between a group’s religiosity and its ability to overcome the problems of collective action inherent in communal life. In the context of this study, examples of costly rituals (in the pandemic context, everyday rituals may turn to risky practices) include efforts to make in-person contact with parishioners or facilitate group gatherings. These behaviors, which can be potentially dangerous to the health of pastors and community members, bring support to the entire group and, through it, group stability, potential enabling the well-being of individuals and the whole community. These ritual mechanisms may buffer stress-induced pressures and positively affect quality of life (Xygalatas et al., 2019). This effect was also confirmed by a longitudinal study from India, which showed that investing in costly rituals is associated with social bonding and subjective health. The same study also showed ritualistic effects on health and emotional processes were stronger in a more traditional context than a cosmopolitan one. This suggests that maintaining kinship and friendship ties in the community plays a key role in subjectively experienced health (Singh et al., 2020).

### Compensatory Control Theory and Double-Edged Sword

The compensatory control theory of religion postulates that religion serves as an external source of control that compensates for uncertainty of control in the inner life of believers (Kay et al., 2008). However, that feeling of control can potentially lead to greater risk behavior (Kray et al., 2008). Some research shows that exposure to God priming can lead individuals to take more significant risks (Chan et al., 2014; Kupor et al., 2015).

The findings of the literature show the diverse influences of religion as a double-edged sword in the context of the COVID-19 pandemic. Religious communities have played both detrimental and beneficial roles in response to the pandemic. Religious groups both accelerated and mitigated the spread of COVID-19 during the early stages of the COVID-19 era (Lee et al., 2021). Therefore, we must sharpen our understanding of which elements of religious practices promote health. Nevertheless, we should also improve our understanding of the different contexts in which religion is likely to have beneficial as well as potentially harmful effects.

The study of religion and mental health has undergone dramatic development in recent decades, but it is necessary to note that these findings and mechanisms of understanding of the social processes by which religion influences mental health and well-being have been examined in relative isolation (Hayward & Krause, 2014). Therefore, it is necessary to look at this phenomenon through an interpretative phenomenological analysis, which can reveal the complex interactions between group and individual facets of religion and their impact on well-being. We want to contribute to an academic understanding of social and psychological phenomena in respect to elaborated processes in religion and their role in improving or degrading individual well-being.

## Research Methodology

### Study Design

This study offers a qualitative understanding of the lived experience of participants who, in their role as an evangelical pastor, were confronted with the COVID-19 pandemic that significantly affected the aspects of their identity derived from encounters with others. In order to uncover the impact of the pandemic on the life, faith, and practice of the church, the method of interpretative phenomenological analysis (Smith & Osborn, 2003; Smith et al., 2009) was used, which has a particular psychological interest in how people make sense of their experiences in the context of their personal and social world (Larkin & Thompson, 2012).

The method has its origins in phenomenology, hermeneutics, and idiography. It emphasizes specific aspects of meaning rather than generalizations. This orientation has its roots in the hermeneutics of Heidegger and Gadamer. Larkin and Thompson (2012, p. 114) show that interpretative phenomenological analysis (IPA) does not test hypotheses and is not usually used to generate theory. This approach requires open-ended research questions that ask about experiences and how people understand them. The knowledge gained through interpretative phenomenological analysis may not provide an explanation of what causes a phenomenon, but it should help to understand what it is like to live in the context of the phenomenon.

IPA is a valuable approach for studying how individuals perceive and make sense of significant experiences that deeply engage their thoughts and emotions. This method is particularly effective when examining events that prompt individuals to reflect on their meaning (Smith, 2019). This approach is particularly appropriate for our research design. The pastors were confronted with difficult situations that placed high demands on them for meaning-making related both to the COVID-19 pandemic and confronting contagion and death, but also to their role as pastors, representatives of God on earth, who must reflect on the meaning of faith in this crisis. Moreover, because of the preservation of physical health, it has been necessary to resort to a transformation of traditional rituals, which raises new questions.

## Research Sample and Procedure

A basic rule for the selection of respondents is to ensure the homogeneity of the research sample. This rule enables researchers to capture the lived experience of the participants as faithfully as possible (Smith et al., 2009). Homogeneity in our case refers to an equal possibility of experiencing the dramatic impact of the COVID-19 pandemic from the position of a parish pastor who is confronted with the infection of loved ones and is forced to change the established order of things in his parish because of anti-epidemic measures. However, respondents vary in age, gender, life story, and experience. Each is a unique and original individual. Brief life descriptions and characteristics of the participants are presented in Table 1. All participants (*n* = 7) in this research are evangelical pastors within one seniority in the Czech Republic. All were confronted with the COVID-19 pandemic and experienced dramatic changes in executing their pastoral duties. These characteristics are created from the most emphasized information that appeared in numerical superiority in the interview. In order to maintain confidentiality and not to associate the stories with a specific person, the parish pastors are identified numerically (Pastor 1–Pastor 7).

**Table 1:** Characteristics of participants

No.	Age	Gender	Q1	Q2	Q3	Q4	Q5	Life story
Pastor 1	61	F	3	4	4	No	Yes	Pastor 1 has been working for 15 years in a medium-sized city (23,000 inhabitants). Her core work is working with families with small children, religious services for the elderly in retirement homes, and Café meetings for the elderly. During the pandemic, she wanted to be close to her parishioners, so she delivered religious services on a bicycle and got a job at the hospital because she felt the need to be close.
Pastor 2	55	M	1	2	4	No	No	Pastor 2 used to be a parish priest in a larger town (90,000 people), but for the past few year he has managed a smaller parish (400 people). He considers personal meetings with parishioners and the community essential in his service. He has been attending to entire families. As well as several mentally ill people for years. In his case, long-hour phone calls often solved the impossibility of meeting with parishioners. In interviews, he emphasized that for him it is crucial that no one in the congregation feels left out.
Pastor 3	41	F	4	–	–	–	–	Pastor 3 manages a parish in a large city (90,500 inhabitants), which she joined a year before the COVID-19 pandemic after maternity leave. At the time of the pandemic, she was a part-time parish priest because she had small children at home. In interviews, pastor 3 emphasized her commitment to work and many hours of preparation, which were even more demanding when transitioning to online services. At the same time, it was uncomfortable for her to talk to someone whom she often did not even see on the screen. She was passionate about creating podcasts, video presentations, and online concerts. During the pandemic, she feared infecting someone, so she often called seniors on the phone.
Pastor 4	51	M	1	1	2	No	No	Pastor 4 has been working as a parish priest in a small village (990 inhabitants) for more than 20 years; at the same time, as a senior, he manages other parishes administratively and participates in meetings of fellow parish priests. He leads groups of children in primary schools, groups of youngsters, but also various home groups. Pastor 4 understands religious services as a meeting of people and therefore did not want to do online broadcasts. He emphasized in-person sessions, even during the pandemic. He focused on personal service to the faithful, delivering his reflections to them in person, which often included a short service at a parishioner’s home.
Pastor 5	64	M	2	4	–	Yes	No	Pastor 5 has been a parish priest for 40 years, of which 30 years he has been pastor in a small village (up to 50 inhabitants), but his district includes another 60 villages. In sum, his community was quite large. For years he managed this village alone or with his wife. He often invested all of his time and often finances into the community. For example, he purchased a minibus to transport children (40) from his funds. He devoted himself greatly to children. His religious education was often conducted more like an interest group (e.g., theater, drawing, music, boating club). He canceled all his children’s and senior groups during the pandemic because he didn’t want anyone to get infected. Pastor 5 did not start online teaching, but he sent his sermons. He became infected with the virus during a meeting of pastors during the Lord’s Supper, drinking from a common goblet. He experienced a very severe case of COVID followed by long-term consequences.
Pastor 6	48	M	3	4	4	No	Yes	Pastor 6 has been a pastor for 13 years; for ten years, he served in a medium-sized city (12000 inhabitants), and now he works in a small village (362 inhabitants). He is married and has four children. The content of his work is primarily the preparation of religious services, “which should come first,” but also taking care of people. He understood the restriction of religious services as a drastic step. During the lockdown, he printed and distributed sermons, published audio recordings, and called people by phone. During the pandemic, he realized that “God is still with us, even when we are in trouble.” He sought support for himself from his wife, his sister, and a church elder. At the height of the pandemic, all his children were at home for online education, and he had to help them; this brought him closer to his family. He suffered severe COVID with bilateral pneumonia, during which he realized the fragility of life and concluded we should be more humble.
Pastor 7	64	M	3	1	2	No	No	Pastor 7 has been a pastor for 24 years, working in a small town (9,000 inhabitants). During the pandemic, he mostly stayed at home and left the apartment only when he went to the store. He canceled visits to people and sent sermons to them by e-mail. He did not understand the pandemic as the “end of the world’, but at the same time, he perceived it as a test of faith. He considered COVID a “worse flu” but felt the need to protect himself.

Q1: I am really scared of COVID-19.	1. I strongly disagree; 2. I disagree; 3. I neither agree nor disagree; 4. I agree; 5.I strongly agree
Q2: I perceived this restriction as a threat to our community.	1. I strongly disagree; 2. I disagree; 3. I neither agree nor disagree; 4. I agree; 5.I strongly agree
Q3: Seeing others participating in the Lord’s Supper during the COVID-19 pandemic makes me feel more connected to the community.	1. I strongly disagree; 2. I disagree; 3. I neither agree nor disagree; 4. I agree; 5.I strongly agree
Q4: You contracted COVID-19 during the Lord’s Supper	Yes/No
Q5: You contracted COVID-19 somewhere else.	Yes/No

The ethical requirements of the research were consulted with the members of the Faculty Ethics Committee before the commencement of the research, and the conduct of the research was found to be unproblematic. The pastors were approached at a very turbulent time when the Czech government issued new measures and restrictions weekly for people to follow. However, this frequent updating and altering of restrictions often led to confusion among the population, where only roughly half understood the restrictions (TN.cz, 2021). It is, therefore, necessary to point out that even the pastors in our study acted according to their best conscience and often consulted their lawyers about their actions. The research participants were first approached by a researcher with ties to the given religious group in question. The pastors then further disseminated information about the possibility of participating in the research among themselves. Prior to the in-person interview, participants were informed about the research through informed consent. They all also consented to the recording and use of the materials for research purposes. Personal consent to publish research data was obtained from all study participants. Pastors were informed that the article would be about sensitive personal information related to COVID-19. Pastors were instructed in the consent that they choose the areas and topics they want to discuss, and they can reject a topic if they find it unpleasant. Although the pastors often acted on the edge of the law, the ethics commission confirmed their incrimination is impossible. The research was based on in-depth semi-structured interviews lasting approximately 45–60 min. The interviews were conducted in the research participant’s preferred setting. The recordings were transcribed verbatim and supplemented with notes identifying the research participants’ emotions and other contextual circumstances.

The participants also filled out a short questionnaire in which they answered questions about the essential attitude and feelings toward COVID-19 pandemic, according to Ahorsu et al. (2020). This short questionnaire was supplemented with questions about experiencing COVID-19 and the relationship between the Lord’s Supper and COVID-19. The critical questions from this questionnaire are listed in Table 1.

### Analysis

A hermeneutic approach is applied in the analysis, giving emphasis to the fact that research cannot begin without some understanding of the “life-worlds of the pastors.” Interview transcripts have been repeatedly read and annotated. In our study, note-taking was conducted by four researchers. Each reading is associated with a new understanding of the whole interview, with new patterns emerging between cases. In the analysis phase, there is some dialogue between the researchers, who each have specific knowledge and data that they acquired reading the interviews (Larkin et al., 2006; Smith et al., 2009).

It is important to avoid short-hand readings and not to approach interviews in a reductive manner. The note-taking itself takes place on several levels which relate to the descriptive, linguistic, and conceptual characteristics of the interview. These characteristics are represented graphically (by underlining, italics, etc.). In order to ensure the maximum number of important meaning-making units and concepts to which the participants ascribed meaning were recognized, line-by-line coding was used. As the number of interviews increased, new themes emerge. Exploratory comments by the researchers were also made at this level of analysis. As shown by Smith et al. (2009), at this stage the narrative flow of the interview breaks down, which is a consequence of the hermeneutic procedure—the original whole of the interview becomes a set of parts that are later combined into a new whole. An example of the annotation process is shown in Table 2.

**Table 2:** Example of note-taking and coding

Transcript	Notes	Code
So, you see, you got it right, because we never stopped singing—into the masks, respirators, which is not much. *Jeez, now I’ve turned us in!* You see*, it’s a good thing, if you ask me*, that I didn’t ban people from singing. I thought that was a basic human right that no one can forbid us. We kept our spacing and *hummed into* our respirators. Quietly, but we never stopped singing. We couldn’t imagine it without singing—praising God, nobody can forbid us. *Not even a pandemic!* A little irresponsibility only	*Admitting to breaking the rules—they consider this rebellion important*; it sets boundaries that cannot be crossed Triple repetition of the word singing *Amplification of emphasis*	Singing—rebellion to protect mental health Human rights Singing as praising God Admission of irresponsibility

During the analysis (Smith et al., 2009), a list of significant notes referring to participants’ experiences of the pandemic and changes in rituals was created using initial noting. In a second step, systematic note-taking led to the identification of key emergent themes. Through abstraction, the researchers identified key superordinate themes related to two meaning-making processes: making sense of physical health and making sense of mental health. The analysis was carried out by three researchers, who compared their results in the final stage and, based on the discussion, compiled a list of the most significant themes and their relationships. The different phases of the analysis are presented in Table 3. The dynamic relationships between the dimensions of physical and mental health experienced by parishioners are described in the discussion section.

**Table 3:** Overview of analysis according to IPA analytical principles Smith et al., (2009)

Initial noting	Emergent themes	Super-ordinate themes	Making sense of
Activities disruption, making phone calls, online communication as a common practice, online Bible lessons, audio recordings, written sermons, distribution of texts, prohibition of assembly, outdoor services	Social distancing	Hygiene measures to protect health	Physical health
Problems interpreting exceptions, refusing to wear masks	Mask wearing		
Absurdity of measures, singing in masks, violation of human rights	Prohibition of singing		
Family visits, rebellion against the measure of the faithful, underestimating the situation	Accepting risk	Attitudes toward risks of infection	
The shock of the pandemic, faith is not protection from disease, fear of contagion	Avoiding risk		
The harmony of science, medicine and faith, reliance on vaccination	Vaccination opinions		
Cups instead of chalices, contagion during the Lord’s Supper, people’s reluctance to go to ritual, criticism of the hygiene of ritual even before the pandemic	Lord’s Supper	Transformation of traditions to protect health	
The online world as an opportunity to reach more people, changes in giving Communion	Services		
The loss of human conversation, uncertainty about communication, the problem of returning to the real world	Mental effects of social distancing	The role of the community and its support	Mental health
“We never stop singing!”, awareness of the importance of meeting—violation of social distancing, mental support for believers despite the risk of contagion	Rebellion to protect mental health		
Cooperation between churches, the importance of informal meetings for problem solving	Community and social support		
People’s interest in the church during a pandemic	Rituals and traditions		
Caring for people, empathy, worrying about people	Solidarity		
Meeting with a psychotherapist, the importance of dialogue for dealing with crisis, burnout prevention	Supervision group	Coping and compensatory control	
The support of colleagues and family	Peer discussion		
Meeting with your own inner self, positive redescription of the crisis, COVID-19 as an opportunity for spiritual growth	The spirituality of solitude and the relationship with God		
The Bible as a guide to the crisis, the mental health significance of the pandemic—recognizing the important aspects of life, the need for faith in society, rallying people to faith during an emergency	Importance of faith	The therapeutic potential of faith	
The competence of the pastor vs. the psychologist, the dangers of the believer’s self-confidence	Limits of faith		

The questionnaires show that the respondents did not feel much fear about COVID-19. Their answers show that only one of the pastors worried about COVID-19, three expressed a neutral attitude, and the other three did not agree that the disease aroused fear in them. Three parish pastors strongly perceived government restrictions as a threat to the community, the others disagreed with this statement, and one parish pastor did not comment on the question. Two parish pastors did not have these feelings, and one did not answer the question. At the time of filling out the questionnaire, three pastors had experienced COVID-19 infection, and one of them had contracted it during the Lord’s Supper.

### Ensuring the Rigor of Research

To ensure research rigor, we adopted a triangulation of methods strategy (Carter et al., 2014) which consisted of supplementing the IPA approach (Smith et al., 2009) with a short questionnaire to gain further information about participants and better understand their emotional experience of the pandemic. This questionnaire focused on respondents’ concerns about COVID-19 in the context of their religious practice or whether they had contracted COVID-19 during a ritual or in other circumstances. The question on pandemic fears was inspired by a standardized questionnaire (Ahorsu et al., 2020). Another important aspect of the research was the researcher triangulation (Denzin, 1978), which was particularly significant during coding. As described, the final phase of coding and processing of the phenomenological analysis notes was handled by three coders who collaborated extensively and clarified the method of analysis before coding began to ensure consistency and comparability of results (Lietz & Zayas, 2010). The relationships between the most significant themes are shown in Table 3.

As Nizza et al. (2021) show, to ensure excellence in IPA studies, it is important to focus on four main indicators of quality, namely: constructing a compelling unfolding narrative, developing a vigorous experiential and/or existential account, making a close analytic reading of participant’s words, and attending to convergence and divergence.

The narrative of our study concerns the confrontation with the COVID-19 pandemic in a religious community, which became more dramatic the moment some members of the seniority were infected. The pastors found themselves in a very complicated situation where they had to protect the physical health of the faithful, but at the same time preserve the values of faith that help them face the crisis and shape its meaning, but also the meaning of faith itself. The participants’ accounts thus helped to develop an experiential account that is very existential in nature.

Within the research, we attempted a close analytic reading of participant’s words. In this regard, the involvement of multiple researchers and their discussion of the ways in which participants make meaning of the experience was important. It was essential to capture the unbiased complexity and dynamics of the experience of the parishioners who had to balance in an unenviable way the emphasis on physical and mental health, while being confronted with existential questions about faith. Their stories and lived experience are not black and white; they display a great humanity in their judgments and decisions about accepting risks.

In terms of convergence and divergence, all of the identified superordinate themes were present in all of the pastors’ descriptions of experience. Talking about physical health, themes of hygiene, transformation of ritual and traditions, attitudes toward risks of infection emerged in all pastors’ accounts, which ultimately led to making sense of illness. As the presentation of the results shows, the cases differ in the intensity of the experience of illness, particularly in the respect that one pastor shared his infection with COVID (pastor 6), while one pastor was able to deeply recontextualize the crisis as an opportunity (pastor 1). Similar superordinate themes relating to mental health emerged in all participants’ descriptions of lived experience. Although pastors touched on the theme of the therapeutic potential of faith, they differed in their assessment of the degree of its importance.

### Coping with COVID-19 in the Context of Physical and Mental Health

This section discusses the cross-cutting themes that emerged in the interviews and indicates the dynamic between maintaining health and maintaining religious practices during the COVID-19 pandemic.

### Lived Experience of Faith and Physical Health

*Hygiene measures.* No parish escaped the necessary hygiene measures, which is why all seven pastors mentioned the changes that occurred and the ways in which they experienced the situation. Pastors implemented the measures but often tried to apply them in a way enables kept the religious community together. What bothered the pastors most was the limited opportunity to meet. As Pastor 3, a 41-year-old enthusiastic parish pastor, describes: “this, I think, was the biggest blow, that people couldn’t meet and chat normally.” The experience of the emotions associated with this dramatic change is aptly described in the following excerpt:

“So, it changed mainly in the inability to meet people, that was probably the most challenging, and then actually during that first wave we absolutely didn’t know how to communicate. Because nowadays we look at it as normal because then we went to all kinds of online gatherings and even online services and online meetings and everything, but up until then we didn’t have anything, so it felt like time was completely stopping.” (Pastor 3)

Although initially, some pastors resisted bringing events online, they began to realize the benefits of connection with remote and hard-to-reach locations or even across the globe:

“We’ve realized that we’ve grown closer with people who otherwise live pretty far away, so now I try to do those online services occasionally after the “normal” ones, so I still do one from my living room for the more distant ones, because that’s what they want. From other churches maybe, and middle generation meetings too. Maybe a friend from England joined, or a brother-in-law from Japan for the online ones, so sometimes we try to keep those contacts going. The online world has opened up possibilities that we didn’t realize.” (Pastor 2)

On the other hand, they were increasingly troubled by the lack of real interpersonal contact connected with a shared experience. One example is the ban on singing, a measure that caused great controversy throughout the country (Aktuálně, 2021). As previous research has shown (Sanal & Gorsev, 2014; Coulton et al., 2015), singing together can reduce the stress and anxiety pastors may experience. This fact was also reflected in the great reluctance of the pastors to stop singing. One pastor described how he dealt with the regulation of restrictions on singing:

“So, you see, you got it right, because we never stopped singing. Into masks, respirators, which isn’t much of a... See, that’s good, if you ask me, I didn’t dare forbid people to sing. I thought that was a basic human right that no one can forbid us. We kept our spacing and hummed into our respirators. Quietly, but we never stopped singing. We couldn’t imagine it without singing. No one can forbid us to praise God.” (Pastor 2)

In contrast, the limitation of singing was seen by another pastor as an opportunity to seek new ways of spirituality.

“And in terms of those changes, for example, in terms of the singing, I think it was a significant shift because we then... we took it as an opportunity for other forms of those services rather than suffering from the fact that it was limited. So, we tended to find interesting, new ways of worship where there was more instrumental music, or there were singers in the choir.” (Pastor 4)

According to government regulations, the possibility of the meeting was limited to a minimum number of participants, in many cases only to members of one household. These restrictions were a drastic disruption to the church. However, for the pastors was most important that the community could continue even in minimal conditions. Pastors mentioned that the church was always open for individual meetings or persons present in the church: “And then I changed it, I did extra children’s services, … because we have a big church, so it wasn’t like it was threatening anyone.” (Pastor 4).

Even the wearing of respiratory masks was not always easy:

“Some didn’t give a damn, like the masks, including a bit of me. I didn’t have any symptoms, then I had fatigue syndrome all winter, for a few months, I thought I’d be done by April sometime, it wouldn’t go on. Now it’s gotten a little better.” (Pastor 5)

*Transformation of ritual and traditions*. The transformation of rituals took place on many levels, from restricting meetings, banning singing, changing the serving of bread at worship to prohibited handshaking. But the “most significant measure is the taking of communion through small individual cups” says Pastor 4. Hygienic measures pushed the parish pastor either to abolish Communion or to transform it:

“… For hygienic reasons, they switched to a slightly different way of celebrating, not from one chalice but from individual cups.” (Pastor 2)

Pastor 1 explains that they first tried to disinfect the large chalice, but also arrived at the small chalices, despite the fact that they raised ridicule among some colleagues:

“Well, my colleagues and I talked about it, some laughed about it, and some just... And it’s true, I’ve already started disinfecting the cup with dental disinfectant wipes. They don’t smell, but they’re good, it says 99% protection... I got that from a doctor. So, we disinfected the chalice that way and were offered those small cups. And then after the lockdown, we started using the little cups permanently.” (Pastor 1)

The transformation of the ritual was perceived very sensitively. At the same time, some pastors tried to keep the ritual from losing its uniqueness:

“I always say that it is not about the form, it is about the substance. I know it’s not nice to have plastic in your hand. That the golden chalice at communion is something different, and there’s a line to the past that goes with it. In some congregations they have cultivated it even more by getting sets of glass chalices to make it look nice. I think it was really thought through so that it wasn’t just a necessity, but that the services would be nice, even the serving...” (Pastor 1)

On the other hand, some pastors found the transformation of the chalice into small cups a good way to modernize the ceremony:

“I’ve experienced other evangelical churches to the west of us, that they don’t consider it some kind of horror, even before the pandemic, to take communion from those cups. So, I would be in favor of keeping it.” (Pastor 6)

*Attitudes toward risks of infection.* The approach to disease risk varied between individual pastors. What they had in common was a desire for continuity of religious practice. Initially, parish pastors were willing to accept more risk in order to preserve the traditional functioning of the community and the traditional values of the church.

“I certainly didn’t see it as the end of the world…..”, “I just always thought it was a worse kind of flu too, that you needed to protect yourself.” (Pastor 7)

The decision to transform rituals and be more vigilant was also driven by personal experience of illness. A number of parish pastors became infected during a seniority meeting. Strong emotions describe Pastor 2, who learned that one of his fellow parish pastors had been infected with COVID-19 and ended up in the intensive care unit:

“But I’ve had people confide in me that loved ones have died, grandmothers, grandfathers have passed away. Not that it’s automatic. The way I was experiencing it, the fear, the great fear, I was also worried that the grief would break someone’s faith…Because I accompany people who are sick, and we know that it’s not going to get better—it’s going to get worse over the years. It’s strange when I think back on it, it was a very strange year and strong, but in those encounters it brought us even more together. We believe we are one body in Christ, one rejoices with the other, but also hurts the other. I’m not saying it works 100%, it doesn’t, but I think it showed up a little bit here. More than we expected. I’ve often worried about different people and wondered why and what will happen now.” (Pastor 2)

Pastor’s 2 story shows how mutuality and group cohesion can help in the most difficult moments:

“It’s brought us even closer together with some people. And I can’t say why some survived, some didn’t, why these. But even the brother who was given no hope, he just survived. He’s going to work now. We just... we were scared, I can’t say we’re some heroes of the faith, I’m not, I’m certainly not, but we were just together, and we bonded with some of them. Then people thank me, and I don’t know what for. I haven’t really done anything.” (Pastor 2)

On the other hand, the pastors tried to protect their congregation:

“At a certain stage we canceled holding services. People were afraid, and I didn’t want to expose them to decision making – if I said: let’s do services, they might come, but at the same time they would be afraid of getting infected. And if they happened to get infected, I would still blame myself that they got infected in church.” (Pastor 5)

All seven pastors took a positive stance on vaccination. They did not regard science as something contrary to faith. The pastors did not rely on God to protect them and considered these ideas foolish. As will be shown in the discussion section, the parish pastors themselves are very aware of these dilemmas and found themselves in very complicated decision-making situations.

*Assigning meaning to illness.* In their years of practice, the pastors have encountered many seriously ill among their fellow community members. Even during the pandemic, each of the seven pastors was confronted with illness and death—whether in the media or in their own experience. These realities raised deep existential and theological questions in most of them. In this link, Pastor 2 mentions: “I then say: But God also created doctors and people who developed respirators…” Pastors reflected on the meaning of illness itself and the whole pandemic. Pastor 6, a father of four, shared his experience as follows:

“Then I also got covid, and a heavy covid at that. I had bilateral pneumonia. So, I realized that it’s not really that much fun, that you can die, and that life is so many times in the balance and it would be terribly easy [to die].” (Pastor 6)

Meaning can also be found in pain and suffering. Pastor 1, who tried to be close to her parishioners as much as possible during the pandemic, says:

“I believe it is. But that it can be somehow cathartic, healing, to realize what is essential for us in life. No one promised us that human life would be pain-free, but that it has meaning. That’s true. So, look for meaning even when it’s hard, Christ is in that.” (Pastor 1)

Here, we see the lived Christian wisdom that comes from the possibility of achieving meaning in spite of suffering and pain. The stories of all seven pastors have shown that this formation of meaning does not lead to resignation, but to a tenacious resistance to conditions that disrupt the practices of their community.

### Lived Experience of Faith, Community and Mental Health

*The role of the community and the effort to maintain it.* All seven pastors mentioned the importance of fellowship. Individually, however, they differed in the ways they tried to preserve its importance. It is the personal encounter, the opportunity for discussion and mutual support that is crucial for church members. Personal contact and closeness, for example, characterized by frequent handshakes, is something without which they cannot imagine the functionality of the community.

“Last spring, when the first wave of the pandemic came, we were kind of hesitant. I didn’t want to go online right away, because that face-to-face encounter is very important to us – and the community in general.” (Pastor 2)

Despite the possible transmission of the disease, some pastors sought to keep the community together. Their behavior had the characteristics of credibility enhancing displays:

“I started working a bit as a Charity volunteer, and I cleaned up in the hospital ... so I got a visit (parishioners) in the hospital ... It was easier in that Charity volunteer T-shirt.” (Pastor 1)

The cost was not only in the form of risky behavior, but also in the form of extreme time investment, but also printed and delivered sermons door-to-door during the lockdown or spending hours on the phone and e-mails:

“Well, I was also forced to call people more because we could not meet in person, so I had those pastoral conversations over the phone.” (Pastor 6)

“[A great deal of pastoral work] actually was done by phone and e-mails, too, so I don’t know how many hours per day I was on the phone.” (Pastor 2)

Some pastors decided to move the meeting to an individual level by visiting individual parishioners’ households. Pastor 4, who thought of religious services as a meeting of people, did not want to do online broadcasts, tried to stay in daily physical contact with parishioners:

“I wrote up some text, thoughts, reflections, copying and distributing to some people, and used it with some visit, sometimes a short communion service at home.” (Pastor 4)

The actual distribution of the texts was very time-consuming. Parish pastors who chose this strategy of keeping in touch with the faithful had to invest a great deal of energy:

“Not everyone had an e-mail, the older people, so I had to prepare services, plus distribute or create a network of people who would be willing to distribute multiplied copies to the others.” (Pastor 6)

Maintaining the community was hence closely linked to a great effort, which was often associated with exposing oneself to the risk of actively visiting many households. Such risky signals during the crisis on the part of pastors may have also indicated the sincerity or depth of their belief and may also increase the credibility of the pastors in the group (Henrich, 2009; Singh & Henrich, 2020).

The pastors thus found themselves once again in a very complicated decision-making situation—the faithful needed their psychological and spiritual support, but at the same time, physical contact created a risk of transmitting the virus. They had to find strategies to compensate for this shortfall.

*Coping and compensatory control.* Just like parishioners, the pastors were also looking to understand the extraordinary situation in their lives. Their support was precisely their religious faith, which compensated for an external source of control for uncertainty in their lives (Kay et al., 2008):

“So, in terms of faith, somehow I didn’t feel that the pandemic could threaten the faith and the spiritual life of those people. I think that maybe on the contrary it could deepen it somehow, because also the solitude or being at home more often and being alone with yourself is something that leads to some experience that is also spiritual. … So I don’t think it has in any way diminished the spiritual life, the faith, or the existential questions of those people, quite the opposite.” (Pastor 4)

The pandemic also led to a deepening of inner questioning that touches on existential issues. Faith can provide believers with support for coping with psychologically challenging situations during a pandemic.

“Faith somehow fortifies a person in those situations in life, in decision making, in the fact that one is not alone. …But for me personally, faith is a kind of relationship, a living process that is affirming and healing on a daily basis” (Pastor 4)

However, according to the pastor, faith is not something that should be singled out over other coping strategies, as different approaches can also be helpful. The pastors themselves, however, find faith to be a dependable strategy for coping with difficult situations. Although some pastors perceived a threat to the religious community and feared that parishioners would lose their faith, their own beliefs gave them a sense of compensatory control (Kay et al., 2008).

The importance of sharing the situation with other parishioners also played an essential role in coping strategies.

“But just the fact that we can talk to these people about the struggle, that we can say, it’s hard, we’re struggling, but we have some hope that carries us through it…I found this terribly important that I can share that with these people.” (Pastor 3)

The importance of dialogue for coping with difficult situations and existential disruptions is also shown in research based on existential hermeneutic phenomenology (Müller et al., 2022). It is the expression of problems via language and the questioning of experienced assumptions that is significant for the positive redescription of crises and the discovery of new coping possibilities. People need to share their experiences in order to experience development (Müller & Kubátová, 2022).

*The therapeutic potential of faith.* All seven pastors touched on the importance of faith and its therapeutic potential in their descriptions of lived experience. Four pastors emphasized this theme the most, while some of them acknowledged that although faith is important, it may not always help.

People of faith can find important support in their faith, particularly those who are struggling with a life trial such as illness. Pastor 2 describes his experience of passing on the faith to old and sick people whom he has been visiting for generations:

“So, I think faith has therapeutic potential. And when I think of the lady in the home for the elderly who had been lying down for years and could only move her mouth. And how she was able to encourage all those around her who were relatively much better off than she was, while mentally they were much worse off and they took turns and she encouraged them... so... yeah, I think that the hope that carries us, that’s not of us, that’s just there, for us, because God loves us all, even though it doesn’t seem like it sometimes, I think that’s what can hold people. And for that I am so grateful.” (Pastor 2)

Although pastors believe that God can protect them to some extent, they realize there are limits because faith may not always help:

“Maybe I wish it were a little bit like that, that faith could sustain a person even in difficult moments. We’ve been through hard things in our family too, sad and tragic things, and so I feel like that faith has helped us a lot. And the way I present it, it can help. It can help, but it doesn’t have to.” (Pastor 5)

This awareness, according to Pastor 5, should be kept in mind even in difficult times such as a pandemic:

“With the pandemic – for me, faith is not such a shield against all evil. I have also experienced that death has not escaped us. Our oldest son was 20 years old... So, I don’t believe that faith protects a person from everything bad, from falling in the mountains or off a cliff, from drowning, from accidents or from pandemics. Many of those people who lived said that in their greatest moments God gave them the strength to get through it, not that He protected them from it.” (Pastor 5)

Other pastors also realize that the power of faith cannot be overestimated. Pastor 4 realizes that even though for him faith is a daily process that heals him, he sees that it is possible to find sanctuary elsewhere: “Alternatively, then, people may look outside of faith for support.” Pastor 6 believes that in specific crises, such as war or pandemics, even non-believers look for something that transcends them. Regarding the therapeutic potential of faith, he speaks as follows:

“I think faith has therapeutic potential. I also wanted to mention that when a person – this is an experience again from that war – was on the front and encountered a threat to his life, the non-believer also started to pray. He also needed something that transcended him. He needed someone to protect him or guide him through that difficult crisis.” (Pastor 6)

Analysis of the interviews showed how important psychological well-being is for community members. However, as the analysis revealed, evangelical pastors found themselves in a very complex decision-making situation. Our qualitative research and focus on lived experience allowed us to understand the inner world of the pastors and the tension between protecting physical health and preserving tradition and mental health. It is this theme that is the focus of the following discussion and summarizes the implications of this unique portrait of difficult pandemic times.

## Discussion—The Dynamics Between Physical and Mental Health

Parish pastors have faced a very difficult situation since the beginning of the pandemic. Analysis of their lived experience revealed that in all narratives there is a dynamic between the lived experience of physical health and the lived experience of mental health that affects not only overall decision making about the future development of the church, but also the health of the members of the community.

In light of this dynamic, the COVID-19 pandemic created a paradox in that those activities which can lead to positive mental health, such as ritual behavior (Lang et al., 2015) and singing in a choir (Sanal & Gorsev, 2014; Coulton et al., 2015), and additionally represent common strategies to achieve group well-being, had a negative impact on physical health by increasing the risk of contracting COVID-19. The mental well-being of the group studied was clearly based on regular meetings and the functionality of the community as a place for mutual social support, shared spiritual activities, and solidarity among church members. These shared activities are, in many ways, more significant than the rigidity of rituals. For example, one pastor mentioned the priority of time meaningfully spent over religious lessons when working with youth. Another pastor mentioned that the psychological support of the group members (even though it may not be very frequent) is the real reason and purpose of the pastor’s work.

The pastors’ sense of identity is deeply rooted in their work with those they provide spiritual and social support. Therefore, as part of the ritual, pastors were more willing to alter the form of the ritual (slicing bread, using small cups) than the social manifestations of the ritual, such as singing together or ritualized handshaking. For these reasons, the lived experience of mental health was significantly prioritized at the onset of the COVID-19 pandemic. Along with that, these signals (CREDS) displayed by pastors in front of the faithful could also strengthen the community and their mutual commitment (Henrich, 2009). The main aim was to ensure the maintenance of the community, which is fundamental to the well-being of the group.

**Fig. 1:**
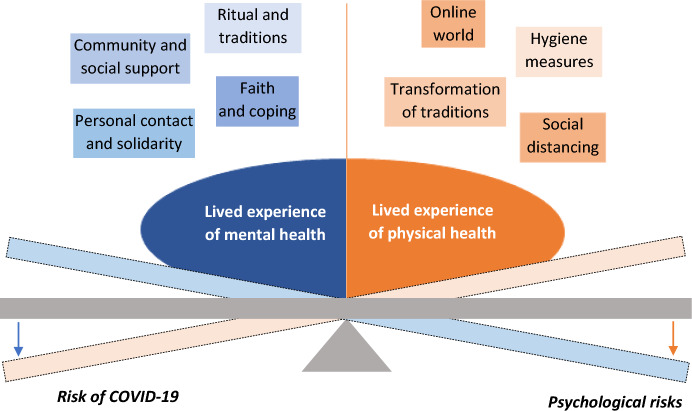
Dynamics between lived experience of mental and physical health

Many hygiene measures aimed at preventing the spread of the disease were put in place by religious groups. In order to protect physical health, the church was willing to retreat from traditional rituals, for example, the use of just one chalice at communion. At the same time, however, they have been unable to refrain from certain activities that maintain the functionality of the community, even though they may be risky for the pastors. Encounters persisted in some parishes, for example, in the form of home services or when one parish pastor visited the sick in hospital. These activities were considered necessary for spiritual, mental and social support for church members, although they may have posed a risk of contagion. The balancing act between the plane of mental and physical health, which presents a significant decision making and ethical dilemma, is illustrated in Fig. 1.

### Study Limitations

An important limitation and consideration of this study is the fact that it was conducted over a period of several months after a key event, the communion ritual under both kinds, where pastors were infected with coronavirus. A fuller understanding of the complexity of interactions between group and individual facets of religion and their impact on well-being, would be better gained through a longitudinal study. Our findings do, nonetheless, offer important insight into the relationship between physical and mental health concerning the three essential elements of religion: ritual, faith, and religious community in a specific pandemic situation.

## Conclusion

This study presents an interpretative phenomenological analysis (IPA) of evangelical pastor’s group strongly affected by the COVID-19 pandemic, revealing the complex interactions between group and individual facets of religion and their impact on well-being. We aim to capture the interaction between physical and mental health concerning the three essential elements of religion: ritual, faith, and religious community in a specific pandemic situation.

The analysis shows that physical health is closely linked to the limitation of ritualized activity, where, despite initial reluctance, there was a fundamental change in the main communion ritual. Pastors were more willing to alter or limit the form of the ritual (slicing bread, using small cups) than the social manifestations of the ritual, such as singing together or ritualized handshaking. The complete interruption of the ritual and the closure of the churches was unacceptable to most pastors, so they moved very quickly to online spaces or small personal communion services.

The study also showed that a central goal of mental health for pastors and the community motivated meetings despite possible infection. Despite the fact that many pastors tried to abide by the announced restrictions, their efforts to maintain and save the community may have enabled the spread of the disease. At the same time, the pastors themselves made extreme efforts during the pandemic, sending credibility enhancing displays to the community (Henrich, 2009), which had a great response, especially in respect to participation in the communion ritual. Thus, it is clear the social aspect played a crucial role in psychological well-being but simultaneously created a paradox of the possible spread of COVID-19 (Lee et al., 2021).

The narratives of the pastors also showed that faith helped them as a kind of therapy and compensatory control (Kay et al., 2008) which functioned as a supporting element in their lives. At the same time, it is important to note that even if a substantial part of the pastor’s community had been affected by a severe COVID-19 illness, supported by their beliefs, they still tried very hard to preserve the religious community despite the possibility of reinfection (Chan et al., 2014).

Our study aimed to show that a pastor’s behavior that, at first glance, seems risky can support psychological health and the preservation of the entire religious group. From the individual interviews, it became clear that the risky signals that the pastors carried out, and which threatened their physical health in times of crisis, bonded the community and, from a longer-term perspective, supported its survival (Henrich, 2009; Sosis, 2000).

This study, however, covers only the period of the greatest spread of COVID-19 and the time immediately after. It would certainly be worthwhile to examine the dynamics between mental and physical health in other pandemic periods, when different conditions may prevail. The group of pastors surveyed felt a significant threat to their religious groups and some even spoke of a possible extinction under the influence of the pandemic. That concern could have activated a dynamic that sought to save the group even at the risk of individual costs (drinking from one goblet) (Sosis, 2000) and via credibility enhancing displays (Henrich, 2009; Singh & Henrich, 2020).

However, subsequently, the pastors found that online broadcasting and other costly activities caused increased participation of believers. In a period where pastors do not feel the threat of extinction, a reduction in costly signals and a decrease of risky behavior might be observed. Other mechanisms might also be found in larger religious groups, where their threat will not be as significant as in case of one of the small local groups of the Evangelical Church in the Czech Republic.

## Data Availability

The datasets generated and analyzed in the current study are not publicly available to honor the individual privacy of the participants but are partially available from the corresponding author on reasonable request.
